# Health Conditions in Older Adults Suspected of Being Maltreated: A 20-Year Real-World Study

**DOI:** 10.3390/jcm12165247

**Published:** 2023-08-11

**Authors:** Hugo Graça, Sofia Lalanda Frazão, Teresa Magalhães, Paulo Vieira-Pinto, Joana Costa Gomes, Tiago Taveira-Gomes

**Affiliations:** 1 Faculty of Medicine, University of Porto, 4200-319 Porto, Portugal; up201607354@edu.med.up.pt (H.G.); smfrazao@icbas.up.pt (S.L.F.); 2 National Institute of Legal Medicine and Forensic Sciences, Largo da Sé Nova, 3000-231 Coimbra, Portugal; 3 Abel Salazar Biomedical Sciences Institute, University of Porto, 4050-313 Porto, Portugal; joanacostabgomes@gmail.com; 4 MTG Research and Development Lab, 4200-604 Porto, Portugal; tmaga@med.up.pt (T.M.); tiagogomes@med.up.pt (T.T.-G.); 5 Center for Health Technology and Services Research (CINTESIS@RISE), Faculty of Medicine, University of Porto, 4200-319 Porto, Portugal; 6 Toxicology Research Unit, University Institute of Health Sciences, Advanced Polytechnic and University Cooperative (CESPU), CRL, 4585-116 Gandra, Portugal; 7 FOREN—Forensic Science Experts, 1400-136 Lisboa, Portugal; 8 Faculty of Human and Social Sciences, University Fernando Pessoa (FCHS-UFP), 4249-004 Porto, Portugal; 9 USF Caravela, Local Healthcare Unit of Matosinhos, Rua da Lagoa, 4460-352 Senhora da Hora, Portugal; 10 Faculty of Health Sciences, University Fernando Pessoa (FCS-UFP), 4249-004 Porto, Portugal

**Keywords:** older adult, maltreatment, health risk factors, health conditions, physical disorders, mental disorders, healthcare professionals

## Abstract

Older adult maltreatment (OAM) is a global problem that has attracted increasing attention due to the ageing population and its severe impact on victim health. Thus, this study aims to analyse the prevalence of certain health conditions in people ≥ 60 years old whom physicians from a local healthcare unit suspected to be victims of maltreatment. The specific objectives are to determine the prevalence rates of health-related risk factors, traumatic injuries and intoxications, mental disorders, and physical disorders. We conducted a real-world, retrospective, observational, and cross-sectional study based on secondary data analyses of electronic health records and healthcare registers of patients at the Local Healthcare Unit of Matosinhos (2001–2021). Information was obtained based on codes from the International Classification of Diseases, codes from the International Classification of Primary Care, and clinical notes (according to previously defined keywords). We identified 3092 suspected victims of OAM, representing 4.5% of the total population analysed. This prevalence is lower than the known rates. We also found that some health risk factors, traumatic injuries and intoxications, mental health disorders, and physical disorders presented higher rates in the suspected victims than among the total population. In this age group, we cannot assume that these health problems are only related to a possible current victimisation process; they could also be associated with adverse childhood experiences or intimate partner violence, among other forms of violence, all of which can lead to cumulative effects on the victim’s health. This evidence increases healthcare providers’ responsibility in detecting and reporting all cases of suspected maltreatment.

## 1. Introduction

According to the World Health Organization (WHO), older adult maltreatment (OAM) is a single or repeated act of commission (abuse) or a lack of appropriate action (neglect) that causes harm or distress to an older adult within any relationship in which there is an expectation of trust or dependence [1,2].

OAM has been acknowledged as an extensive and severe problem and a violation of human rights. It is associated with devastating individual consequences and social costs and urgently requires attention from social welfare agencies, healthcare systems, policymakers, and the entire community [3].

OAM may manifest itself in different forms [4,5]: (a) abuse (e.g., psychological, physical, sexual, or financial); (b) neglect (e.g., emotional and physical); (c) self-neglect (behaviour that threatens one’s health or safety; it includes a situation in which a mentally competent person who understands the consequences of his or her decisions makes a conscious and voluntary decision to engage in acts that compromise his or her safety as a matter of personal choice); and (d) abandonment (desertion of an older adult by an individual who agreed to care for him or her or by the person who has his/her physical custody).

The prevalence of OAM has grown in parallel with the global increase in the ageing population [6,7]. The prevalence estimates vary widely, depending on concepts, studied populations and settings, and screening tools. A report by the WHO (2017) stated that the global prevalence of OAM is 15.7%, which means that 1 in 6 people aged 60 or older has experienced some form of maltreatment in community settings [8]: (a) 11.6% have experienced psychological abuse; (b) 6.8% have experienced financial abuse; (c) 4.2% have experienced neglect; (d) 2.6% have experienced physical abuse; and (e) 0.9% have experienced sexual abuse.

OAM is increasingly being recognised as a major global public health problem, as it may cause potential or concrete harm to the health and well-being of victims [1,2,3,9]. The immediate effects of OAM may include acute injuries, ranging from bruises to more disabling consequences (e.g., bone fractures or brain trauma injuries), and some health disorders, such as persistent physical pain and soreness, poor nutrition and dehydration, sleep disturbances, and susceptibility to new illnesses. When approaching a patient with injuries, the physician must consider all the possible differential diagnoses, such as [10]: (a) traumatic injuries (accident, intentionally inflicted by a third person, self-inflicted, and iatrogenic); (b) pathological injuries (signs related to an underlying disease or condition, such as spontaneous ecchymosis); and (c) morphological conditions (e.g., Mongolian blue spots, melanocytic nevus, and some malformations).

The long-term outcomes related to OAM may include adverse mental effects, such as depression and self-harm behaviours (including suicide) [11,12,13], impaired physical health, increased previous morbidity, and premature mortality [1,6].

In these cases, we must always bear in mind that older adults carry with them a long history that may include violence experiences, as well as diseases and trauma suffered during their lifetime.

**Figure 1:**
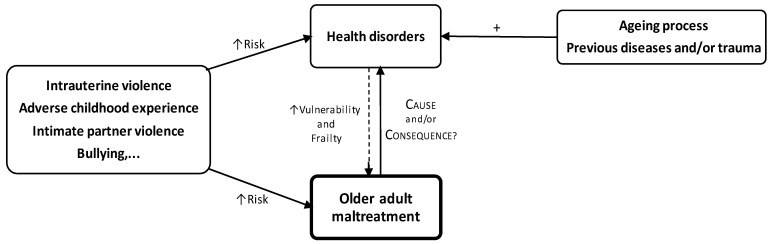
Prior exposure to adverse experiences, health disorders, and OAM.

In most cases of interpersonal violence (especially domestic violence), the adverse event is not isolated. The violence often starts very early and continues throughout the person’s life and may result in a cumulative exposure to violence and cumulative negative health outcomes, particularly for older adults [14]. Each episode of maltreatment or other kind of violence inflicted on a person, regardless of his or her age group, may have a negative impact on his or her health in the short and long term [15]. Violent episodes and the resulting health disorders increase the victim’s vulnerability, placing the individual at a heightened risk of revictimisation and further victimisation in later life [16,17,18]. Thus, the victim can be consistently at an increased risk of violence throughout his or her lifetime [19] because violence can trigger a cascade or snowball effect that perpetuates itself throughout the victim’s life. Prior exposure to violence includes, among others [16] (Figure 1): (a) intrauterine violence (e.g., intimate partner violence against a pregnant woman); (b) adverse childhood experiences (e.g., psychological, physical, and sexual abuse, neglect, abandonment, witnessing abuse, household dysfunction, parental separation, or divorce); (c) intimate partner violence (e.g., physical, emotional, psychological, or sexual); and/or (d) bullying.

Furthermore, older individuals are experiencing the ageing process, which is associated with a progressive reduction in functional physiological reserves leads and deterioration of multiple body systems; therefore, these individuals have a low resistance to stressors, an increase in vulnerability, and a worsened response to adverse events, which is also associated with several health disorders [20].

Thus, some of the health disorders resulting from violence, from the ageing process, or from previous morbid or traumatic situations increase the vulnerability and frailty of the victim, which also contributes to heightening the risk of maltreatment (Figure 1). Thus, it is challenging to determine whether the aetiology of some health disorders is only associated with OAM, with lifelong exposure to violence, or to age-related morbidity [1].

The public health sector is directly concerned with OAM, not only because of its profound effects on the victim’s health and healthcare services but also because of the significant contributions that healthcare professionals can and should provide to identify and report suspected cases and reduce their impact [21].

Often, an emergency department visit represents a unique but usually missed opportunity to identify potential OAM and initiate an intervention; this may be the only time an isolated and vulnerable older adult leaves his or her home [22]. Additionally, in the emergency department, a patient is typically evaluated by professionals from different areas who are able to observe, examine, and interact with him or her, thus yielding a significant potential to detect any sign of abuse or neglect [23].

Primary healthcare professionals are also in a privileged position to detect and manage cases of suspected OAM because they share a sustained partnership with the patient, characterised by support, empathy, co-participatory communication, mutual trust, and a holistic approach to him or her [24,25].

Therefore, in the Portuguese case, a local healthcare unit (LHU), which includes hospitals with emergency departments and primary healthcare facilities, is a critical point in the early identification and management of suspected maltreatment cases, especially in older adults.

This study aims to analyse the prevalence of health conditions in people aged 60 years or older, whom physicians from an LHU suspected to be victims of maltreatment. The specific objectives are to characterise the prevalence rates of: (a) health risk behaviours; (b) traumatic injuries and intoxications; (c) mental health disorders; and (d) physical disorders.

## 2. Materials and Methods

This is a real-world, retrospective, observational, and cross-sectional study conducted in an LHU. The methodology is similar to another study conducted in the same LHU and in the same period [13]. This study was based on secondary data analyses of electronic health records (EHRs) and healthcare registers for patients at the LHU of Matosinhos (LHUM). The LHUM is one of the 8 Portuguese local healthcare units, located in northern Portugal, and includes 1 hospital (Pedro Hispano Hospital) and 14 primary care centres (11 family healthcare units and 3 personalised healthcare units). It provides primary, secondary, and tertiary healthcare to an urban population of approximately 172,669 inhabitants and patients from other locations and institutions.

### 2.1. Participants

The inclusion criteria were as follows: men or women (a) 60 years of age or older; (b) suspected of being a victim of maltreatment by an LHUM physician; (c) part of the resident population under the care of LHUM; and (d) having at least one appointment with a primary care physician within the 3 years preceding the index date. The last data lock point was 10 March 2023, and data were collected from 1 January 2001 to 31 December 2021.

The results were categorised based on the information source. Thus, three groups were defined: (a) G1, corresponding to the information obtained from clinical codes and clinical notes (overlapping cases were excluded); (b) G2, including information obtained from clinical codes only; and (c) G3, including information obtained from clinical notes only.

### 2.2. Variables

We considered the following variables, among others: (a) sex; (b) age; (c) health risk factors; (d) traumatic injuries and intoxications; (e) mental health disorders; and (f) physical disorders. These variables were defined by the researchers using a group of selected keywords. Researchers evaluated the keywords before incorporating them into the analytical code for further processing. The rationale behind selecting the keywords was grounded in the most commonly used terms to describe such situations within the clinical setting of the LHUM. The classification of data related to variables was based on the: (a) International Classification of Diseases (ICD-9 and ICD-10) and (b) International Classification of Primary Care (ICPC-2)

### 2.3. Data Sources/Measurement

The LHUM has over 20 years of electronic health record (EHR) data, which includes comprehensive information for every patient. Data were extracted from the computerised medical records of LHUM. All patients who met the inclusion criteria were enrolled. All the data analysed had already been recorded in the database at the beginning of this study. No samples were taken as we analysed the total patient population at LHUM, matching our eligibility criteria.

Considering that this is a study of databases with an eligible population numbering in the hundreds of thousands, informed consent was not feasible (subparagraphs (i) and (j) of Article 9° of the Portuguese Total Data Protection Regulation). Data access for analysis was granted after approval by the Ethics Committee (No. 72/CES/JAS of 16 September 2022) and the data protection officer of the LHUM (No. 85/CLPSI/2022 of 5 January 2023). All data processing and analysis were exclusively conducted using analytic programs developed explicitly for this purpose and sent for execution on LHUM servers. There was no data extraction outside the LHUM and no direct contact by the researchers. As an additional security measure, the LHUM’s Information Technology Department identified the processed data following the Health Insurance Portability and Accountability Act (HIPAA) safe harbour standard guidelines before executing the analytic code.

### 2.4. Bias

Since OAM is generally under-recognised and under-reported by healthcare professionals, we consider omission bias to be the main source of bias for this study. Therefore, this study is susceptible to the risk of prevalence underestimation. To address this bias, we employed extensive inclusion criteria and no exclusion criteria, and we used a twofold approach, considering: (a) all relevant ICPC-2, ICD-9, and ICD-10 codes ever registered in all levels of care and (b) all relevant keywords ever written by clinicians in the electronic health records. The authors did not identify any additional potential sources of bias in this study.

### 2.5. Statistical Methods

The researchers performed a descriptive analysis without formal comparisons or other statistical analyses. For all variables, relative and absolute frequencies were reported. The percentage of null values (ø) was calculated for all variables that were computed with information besides diagnosis codes (data not shown).

## 3. Results

We identified a total older adult population of 68,094 individuals, with a mean age of 73 years, 56.1% of whom were female (*n* = 38,482).

**Figure 2:**
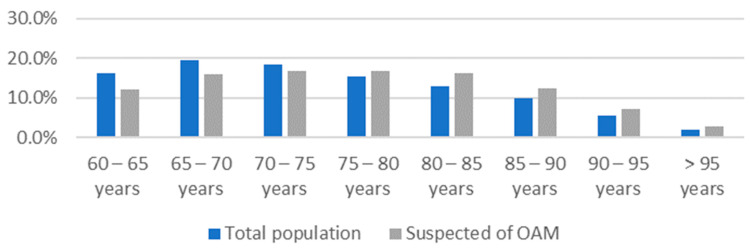
Age distribution of the total population and suspected of OAM.

In G1, the prevalence of suspected cases of OAM was 4.5% (Table 1); the mean age of suspected victims was 76 years (with most between 75 and 80), and the proportion of females was 56.6% (*n* = 1750). Figure 2 shows the age distribution of the total older adult population and the suspected victims of OAM; among individuals aged at least 75 years, the percentage of suspected cases of maltreatment is higher than the percentage of the total older adult population in the same age group.

Table 2, Table 3, Table 4 and Table 5 shows the rates of substance consumption, traumatic injuries and intoxications, mental health disorders, and physical disorders for the total population and the suspected victims of OAM in G1. The tables also show the ratio of the assessed percentage rates of the health conditions between the group of suspected OAM, regarding G1, and the total population (G1/Total). This ratio demonstrates the magnitude of the increase in the rates of health outcomes for the suspected victims compared to the total.

Some other health conditions that were studied are not included in these results because the number of cases was not sufficient for this analysis.

## 4. Discussion

In this study, we found that only 4.5% of the total population analysed was suspected by physicians to be a victim of maltreatment, considering the available information.

The suspected cases of OAM present higher rates of some health risk factors, as well as higher rates of traumatic injuries, intoxications, and mental and physical disorders, than the total population. According to the literature, continued exposure to maltreatment and other contextual stressors are associated with chronic hyperactivation of the hypothalamic–pituitary–adrenal (HPA) axis [26,27]. The HPA axis is the primary system that mediates the biological response to stress, and its chronic dysregulation results in increased inflammatory processes, hormonal changes (such as increased production of cortisol, decreased expression of its receptors, and increased corticotrophin-releasing factor), and further disruption of the neuroendocrine and immune system [27,28,29]. Additionally, HPA dysregulation can trigger some genetically programmed and latent disorders [26,29]. All these alterations play an important role in the pathophysiology of some physical, mental, and other conditions/disorders associated with OAM. However, taking into account the considerations made regarding the pathophysiological mechanism described in Figure 1, the results of our study about the health conditions and health risk factors found in OAM suspects can: (a) be interpreted as causes or consequences of maltreatment or both, and (b) also result from previous maltreatment together with the current.

### 4.1. Risk Factors

Current evidence has described some factors that increase the risk of OAM. Identifying these risk factors is imperative to increase awareness of the problem and properly track older adults at higher risk.

The risk factors for OAM, as well as other forms of interpersonal violence, can be explored regarding an ecological model. This model considers the complex interplay between the victim–abuser dyad, the community, and societal factors. It allows us to understand the multifaceted nature of OAM, the range of factors that put older adults at higher risks, and how all those factors are deeply interconnected [21].

The individual risk factors related to the victim include [3,8,11,30,31,32,33,34,35]: (a) female sex; (b) older age; (c) functional dependency/disability; (d) reduced cognitive status and mental health disorders; (e) substance consumption; (f) prior victimisation; (g) low income; and (h) financial dependency.

In our study, we were only able to identify the following demographic risk factors, in line with the literature: (a) older age (up from 75 years) was associated with higher rates of maltreatment (Figure 2); (b) poverty rate was 1.9 times higher in suspected OAM cases; and (c) alcohol consumption rates were 1.9 times higher (Table 2).

However, the rates of alcohol consumption were much lower than expected, both for the total population and the suspected OAM. We believe that there may be an underlying under-registering situation, partly explained by the recent inclusion (2014) of the codes regarding substance use (tobacco, alcohol, and drugs) on the list of primary healthcare monitoring indicators.

Other victims’ risk factors were not available for this analysis.

### 4.2. Traumatic Injuries and Intoxications

There are some features found in a physical exam that physicians should be aware of, considering that they may increase the index of suspicion of intentionally inflicted injuries, namely [36]: (a) unexplained fractures; (b) bruising at unusual locations; (c) burns in patterns inconsistent with unintentional injury or with the explanation provided (e.g., multiple burns; bruises on the abdomen, neck, posterior legs, or medial arm); (d) patterned injuries (e.g., hand slap or bite marks); (e) ligature marks or scars around wrists, ankles, or neck suggesting inappropriate restraint; (f) subconjunctival or vitreous ophthalmic haemorrhage; (g) traumatic alopecia or scalp swelling; (h) evidence of sexual abuse and intraoral soft tissue injuries; (i) decubitus ulcers, unless related to unavoidable health decline; (j) dehydration; (k) malnutrition and medically unexplained weight loss; and (l) unusual delay in seeking medical attention for injuries.

Establishing the medico–legal aetiology for these injuries is not straightforward, and sometimes it may remain unknown [9]. Thus, physicians should know how to approach and communicate with suspected victims, how to identify risk factors for OAM, and how to perform adequate ancillary exams and request specialised advice (e.g., from forensic doctors) [10]. These cases can be even more complex, as injuries associated with maltreatment are not limited to those intentionally inflicted by a third person, and victimisation itself increases the risk of other injuries, such as accidental [37] and self-inflicted injuries [38].

We cannot determine the medico–legal aetiology of the traumatic cases found in this study. Still, we can affirm that the traumatic injury rate was higher in the suspected OAM group than in the total population. The rates of bone fracture and bone dislocation were 1.4 and 1.9 times higher, respectively. The same was found to be true for superficial injury, open wound, burn, and crushing injury (1.8, 1.5, 2.0, and 1.7 times higher, respectively) (Table 3). Additionally, we noticed that the physicians recorded more frequent cases of bone fractures and deep injuries compared to superficial injuries, while it is known that superficial injuries are more frequent in this population [39]. This may be related to the higher valorisation given to more severe injuries. However, it is important to emphasise that less serious and superficial injuries are frequent red flags of maltreatment that also deserve healthcare professionals’ attention [39] and must be registered.

OAM can also involve the administration of inappropriate substances, inappropriate doses of medications, or failure to monitor drug therapy, which contributes to an increased risk of intoxications [40]. Although intoxications are mostly unintentional in older adults, there is an increased prevalence of intentional intoxications in the victims of OAM compared to their non-maltreated peers [41], which is in line with our findings (the intoxication rate was 1.7 times higher in the suspected victims—Table 3). Additionally, in these cases, we were not able to determine its medico–legal aetiology.

### 4.3. Mental Health Disorders

OAM, in all its forms, has a profound impact on victims’ mental health. This may range from an immediate and acute outcome to a well-established mental health condition resulting from continued exposure to maltreatment, which worsens over time and may last long after the event has stopped [42]. Maltreated older adults are more likely to experience [1,33,38,42,43,44,45]: (a) major psychiatric disorders; (b) major depression and depressive disorders; (c) dementia; (d) memory disorders; (e) sleeping disorders; (f) eating disorders; (g) psychological distress (e.g., loneliness, fear, grief, anger, helplessness, sadness, and anxiety); (h) self-destructive behaviours (e.g., self-inflicted injuries, suicidal ideation, suicide attempts, and suicide); (i) loss of self-confidence and self-esteem; (j) isolation; and (k) social dysfunction.

It is known that the progressive loss of cognitive abilities associated with ageing is explained by several factors, including changes in brain plasticity [20]. Furthermore, OAM is a traumatic event that can trigger or accelerate senile evolution due to decreased neurocognitive adaptation capacity. Consequently, cognitive changes such as impaired consciousness, confusion, altered attention, memory, thought, and perception may appear [46]. These changes can also trigger neuropsychological dysfunction, which may progress to pseudodementia or dementia, and reveal previously unknown mental health disorders [20,46,47], as well as maltreatment cases.

In the present study, we found, in accordance with the literature, that most of the mental health disorders appeared to be higher in the suspected OAM cases than in the total population (Table 1), namely: (a) major psychiatric disorders (1.5 times higher); (b) posttraumatic stress disorders (1.5 times higher); (c) dementia, vascular dementia, and Alzheimer’s dementia (2.0, 2.6, and 1.8 times higher); and (d) suicidal ideation (2.0 times higher). The rates of sleep and memory disorders, psychosocial stress, and social deprivation were also higher in the suspected victims.

We did not include depression disorders in this study because there were very few recorded cases, either for the total population or victims of OAM. One reason for this situation is that physicians might record some depressive disorders inaccurately by including them in a broader and less specific diagnosis (we should note that antidepressant intake presents a rate of 1.3 in OAM suspected persons—Table 3). Additionally, the results reported that suspected OAM has a higher rate of medication consumption compared to the total population, namely, antipsychotics and sedatives, which were 1.7 and 1.3 times higher, respectively (Table 3).

All these mental disorders contribute to a deterioration in health and functional status, leading to a loss of independence and autonomy and, consequently, to a decrease in quality of life due to exposure to maltreatment.

### 4.4. Physical Disorders

Assessing physical disorders associated with maltreatment in older age is a particularly challenging task, considering that this age group has a higher burden of morbidity associated with the ageing process and previous morbidity in life. Studies evaluating health conditions related to OAM have placed slightly greater emphasis on psychological or emotional domains than on physical disorders [42].

Nevertheless, the current literature shows that exposure to maltreatment has enormous repercussions in older age, and the physical health outcomes are very wide ranging [48]. They may be either new conditions or exacerbations of an existing one and include [33,37,42,49,50]: (a) metabolic disorders (e.g., obesity, diabetes, dyslipidaemia, metabolic syndrome); (b) cardiocerebrovascular disorders (e.g., heart failure, hypertension, atherosclerosis, acute myocardial infarction, stroke); (c) inflammatory disorders (e.g., rheumatoid arthritis, lupus, asthma, chronic obstructive pulmonary disease); (d) genitourinary disorders (e.g., chronic kidney disease, urinary tract infection); and (e) general physical symptoms (e.g., chronic pain, headache).

Our findings corroborate and support the existing evidence, given that the evaluated physical health disorders were higher in the suspected cases of OAM than in the total population (Table 5). Regarding metabolic disorders, we found that type 2 diabetes, metabolic syndrome, and non-alcoholic fatty liver disease were 1.3, 2.0, and 1.1 times higher, respectively; the prevalence of cardiovascular disorders was also higher, namely, myocardial infarction, stable angina, unstable angina, transient ischaemic attack, and latest stage of heart disease (1.8, 1.7, 1.4, 1.4, and 2.1 times higher, respectively); and the suspected OAM patients had higher rates of cerebrovascular events, specifically, ischaemic and haemorrhagic stroke (1.9 and 2.9 times higher, respectively). Also, the rates of chronic kidney disease, urinary tract infection, and inflammatory disorders increased.

Notably, as stated before, the incidence of cerebrovascular events was much higher in individuals with suspected OAM. This is a reason for concern, considering that in the literature there has been an association between stroke (both haemorrhagic and ischaemic) and traumatic brain injury, which can, ultimately, be associated with maltreatment [51,52].

### 4.5. The Role of Health Professionals in OAM

In our study, we found that the rate of suspected cases of OAM during the analysed period was 4.5% in G1 (Table 1). This value is lower than that reported in the current literature: (a) 15.7% worldwide [8]; (b) 15.4% Europe [8]; and (c) 23.9% Portugal [53] (the second-most ageing country in Europe [54]). To contribute to this prevalence underestimation, we note that physicians from the LHUM under-registered the cases of OAM in the available documents, perhaps not only because most of the cases were not detected but also because they may have had doubts about the diagnosis. We also consider that there could be additional information on other suspected victims in a confidential register that, for reasons of medical privacy, we could not access.

In fact, professionals point out some constraints that limit their ability to identify and report maltreatment, namely [55,56,57]: (a) limited awareness on the subject; (b) under training and insecurity on how to address a suspected victim; (c) fear of offending the patient and that they might deny the maltreatment; (d) fear of risking their relationship with the patient; (e) concern that they may aggravate the maltreatment situation; and (f) some organisational limitations (e.g., limited time for each appointment; privacy difficulties; lack of protocols on how to manage suspected or confirmed cases of OAM).

Other aspects thought to be implicated in the under-registering rates of OAM may be related to patient disclosure, including [55,57]: (a) vulnerability; (b) social isolation; (c) feelings of shame, stigma, self-blame, and embarrassment; (d) fear of retaliation and escalation of maltreatment; (e) dependency on the perpetrator; (f) emotional attachment to the perpetrator and fear of jeopardising their relationship; (g) fear of being relocated to a nursing home or long-term care facility; and (h) concern about legal implications for the perpetrator.

We found that physicians from the LHUM more often recorded suspected cases of OAM using clinical notes (*n* = 2478) than clinical codes (*n* = 638), considering G3 and G2, respectively (Table 1). This situation may be related to physicians not being familiar with the use of clinical codes for recording this kind of violence.

However, healthcare professionals play an important and unique role in identifying, reporting, and managing suspected victims of maltreatment. In addition, these victims have higher hospitalisation and healthcare-seeking rates than the general older adult population [37,42], which increases the likelihood of detecting these cases in a healthcare facility. They go to healthcare facilities, not only for healthcare assistance but also for health shelters. The victims might have a trusting relationship with the healthcare professional, particularly in primary care services, which is an important factor that allows them to feel safe and more likely to report any maltreatment [57]. Additionally, primary healthcare facilities benefit from outpatient visits, especially when dealing with frail older adults, which is an opportunity to assess the patient in his or her real social and familial context and look for any sign that might suggest a maltreatment risk [6].

Thus, it is important to promote awareness among health professionals regarding the importance of properly identifying and reporting suspicions and the role they play in this process. Identifying and reporting these cases are important to an adequate intervention to protect and treat the victim’s health. It will allow individuals to act preventively and end the cycle of violence, reducing its impact on the victim’s mental and physical health and avoiding premature mortality. We also believe that a patient’s history of exposure to an episode of maltreatment or any other adverse experience since intrauterine life, in childhood or even in later life, should be actively explored and recorded in his or her past medical history by health professionals. It is an opportunity to keep track of the pattern of polyvictimisation (past or current), prevent perpetuated exposure to violence, and treat previous trauma.

In Portugal, healthcare professionals share a close relationship with OAM, having legal and ethical responsibilities. This is because intrafamilial violence and violence perpetrated by a caregiver are considered public crimes. This implies that all governmental employees, including healthcare professionals of the National Health Service (NHS), are legally obliged to report the crimes that they become aware of in the course of their duties or because of them, whether or not the victim agrees (article 152 and 242, Portuguese Penal Code). Furthermore, the Code of Medical Deontology of the Portuguese Medical Association stipulates, in its 27th article, that physicians must take the necessary actions to protect the health of an older adult in the context of maltreatment, which includes reporting the suspicion to the authorities (*Diário da República, 2.ª série - n.° 139, 2016*). However, even with these legal obligations, physicians often do not report suspected cases of maltreatment that they become aware of, as stated before.

### 4.6. Limitations of This Study and Further Research

The major limitation of this study is the bias related to the under-registration of suspected cases of OAM by physicians in the available information. We admit that there could be additional confidential information that was, therefore, not possible to access.

This was a cross-sectional and retrospective study; thus, we could not establish a cause–effect relation between the health disorders evaluated and OAM, and we were not able to determine when the victimisation process started.

Furthermore, the results did not allow us to establish a differential diagnosis of the medico–legal aetiology of injuries and intoxications, and no further discussion could be made on this relevant topic.

In future studies, our aim is to include a greater number of healthcare units in Portugal and collaborate with research groups from other European countries to perform more comprehensive statistical analysis and compare variables. We also intend to perform longitudinal studies.

## 5. Conclusions

Considering the results we obtained from this real-life study, we outline the following conclusions:

1. At the LHUM, between 2001 and 2021, 3092 older adults aged 60 years or more were identified as suspected victims of maltreatment, which represents only 4.5% of the total older adult population analysed (*n* = 68,094);
2. Physicians at the LHUM more often record suspected victims of OAM using clinical notes than clinical codes;
3. Suspected cases appeared to increase with age;
4. Considering the health risk factors evaluated, suspected victims of OAM had higher rates of alcohol consumption;
5. The risk of OAM appeared to increase with poverty;
6. Suspected victims of OAM had more health problems than the total older adult population at the LHUM over the same period. Compared to the total population, the suspected victims of OAM had higher rates of:
  (a) Traumatic injuries, such as bone fractures and dislocations (1.4 and 1.9 times higher, respectively), and superficial injuries (1.5 times higher);
  (b) Intoxications (1.7 times higher);
  (c) Mental disorders, such as major psychiatric disorders, posttraumatic stress disorder, and dementia (1.5, 1.5, and 2.0 times higher, respectively), as well as other mental health disorders, namely, sleep disorders, memory disorders, psychosocial stress, and social deprivation (1.3, 1.7, 1.2, and 1.9 times higher, respectively);
  (d) Suicidal ideation (2.0 times higher);
  (e) Medication consumption, including antidepressants, antipsychotics, anxiolytics, and sedatives (1.3, 1.7, 1.1, and 1.3 times higher, respectively);
  (f) Cardiovascular disorders, such as myocardial infarction, stable angina, unstable angina, transient ischaemic attack, and heart failure (1.8, 1.7, 1.4, 1.4, and 2.1 times higher, respectively);
  (g) Cerebrovascular events, namely, ischaemic and haemorrhagic stroke (1.9 and 2.9 times higher, respectively);
  (h) Chronic immune inflammatory disorder and asthma (1.6 and 1.3 times higher, respectively);
  (i) Other physical disorders included metabolic (type 2 diabetes and non-alcoholic fatty liver disease, which were 1.3 and 2.0 times higher, respectively), respiratory (chronic obstructive pulmonary disease, which was 1.6 times higher), and genitourinary disorders (chronic kidney disease and urinary tract infection, which were 1.5 and 1.4 times higher, respectively).

These health problems can be either causes or consequences of exposure to violence. It is also impossible to know if they are only related to current victimisation or associated with previous exposure to violence or other adverse conditions.

The under-registration and likely under-reporting of suspected cases of maltreatment is particularly alarming in the older adult population, and it has a substantial repercussion on the victim’s protection and health. This evidence increases healthcare providers’ responsibility in detecting and reporting all cases of suspected maltreatment.

## Figures and Tables

**Table 1:** Older adult population observed at LHUM (2001–2021)—*n* (%).

Total Older Adult Population	Suspected of OAM		
	G1 (Codes + Clinical Notes)	G2 (Codes)	G3 (Notes)
68,094 (100)	3092 (4.5)	638 (0.9)	2478 (3.6)

OAM, older adult maltreatment; G1, group 1, information obtained from codes and clinical notes, excluding the overlapping cases; G2, group 2, information obtained from codes only; and G3, group 3, information resulting from clinical notes only.

**Table 2:** Substance consumption—*n* (%).

	Total Population	G1	Ratio
Tobacco	5205 (7.6)	247 (8.0)	1.1
Alcohol	1596 (2.3)	111 (3.6)	1.6

**Table 3:** Traumatic injuries and intoxications—*n* (%).

	Total Population	G1	Ratio
Bone fracture	30,980 (45.5)	1924 (62.2)	1.4
Open wound	22,027 (32.4)	1468 (47.5)	1.5
Intoxication	14,816 (21.8)	1160 (37.5)	1.7
Burns	5542 (8.1)	503 (16.3)	2.0
Bone dislocation	4776 (7.0)	411 (13.3)	1.9
Superficial injury	4743 (7.0)	409 (13.2)	1.8
Crushing injury	1181 (1.7)	88 (2.9)	1.7

**Table 4:** Mental health disorders and related medication consumption—*n* (%).

	Total Population	G1	Ratio
Major psychiatric disorder	29,381 (43.2)	1958 (63.3)	1.5
Psychosocial stress	17,417 (25.6)	977 (31.6)	1.2
Sleep disorders	10,302 (15.1)	591 (19.1)	1.3
Dementia	5898 (8.7)	541 (17.5)	2.0
Alzheimer’s	1148 (1.7)	97 (3.1)	1.8
Vascular	882 (1.3)	105 (3.4)	2.6
Memory disorders	4614 (6.8)	353 (11.4)	1.7
Social deprivation	1022 (1.5)	88 (2.9)	1.9
Posttraumatic stress disorder	147 (0.2)	10 (0.3)	1.5
Suicide ideation	100 (0.2)	12 (0.4)	2.0
Anxiolytics	44,280 (65.0)	2283 (73.8)	1.1
Antidepressants	31,707 (46.6)	1811 (58.6)	1.3
Sedative	19,085 (28.0)	1100 (35.6)	1.3
Antipsychotics	12,146 (17.8)	957 (31.0)	1.7

**Table 5:** Physical disorders—*n* (%).

	Total Population	G1	Ratio
Metabolic syndrome	62,958 (92.5)	2998 (97.0)	1.1
Type 2 diabetes	29,412 (43.2)	1706 (55.2)	1.3
Hypercholesterolemia	24,033 (35.3)	1031(33.3)	0.9
Obesity	14,407 (21.1)	621 (20.0)	0.9
Non-alcoholic fatty liver disease	2440 (3.6)	233 (7.5)	2.0
Cardiovascular disease	54,884 (80.6)	2784 (90.0)	1.1
Hypertension	47,612 (70.0)	2225 (72.0)	1.0
Structural heart disease	14,014 (20.6)	1079 (34.9)	1.7
Atherosclerotic disease	13,981 (20.5)	1082 (35.0)	1.7
Stroke	9183 (13.5)	800 (25.9)	1.9
Ischaemic	7621 (11.2)	685 (22.2)	1.9
Haemorrhagic	955 (1.4)	112 (3.6)	2.6
Atrial fibrillation	8429 (12.4)	716 (23.2)	1.9
Microvascular disease	5163 (7.6)	396 (12.8)	1.7
Angina, stable	4554 (7.0)	318 (10.3)	1.7
Angina, unstable	2189 (3.2)	142 (4.6)	1.4
Peripheral artery disease	4339 (6.4)	324 (10.5)	1.6
Heart failure (latest stage)	3866 (5.7)	374 (12.1)	2.1
Myocardial infarction	3619 (5.3)	288 (9.3)	1.8
Transient ischaemic attack	1001 (1.5)	65 (2.1)	1.4
Early heart disease	749 (1.1)	48 (1.6)	1.5
Chronic obstructive pulmonary disease	4970 (7.3)	355 (11.5)	1.6
Asthma	3257 (4.8)	188 (6.1)	1.3
Chronic immune inflammatory disorder	1694 (2.5)	122 (4.0)	1.6
Chronic kidney disease	14,895 (21.9)	1010 (32.7)	1.5
Urinary tract infection	2303 (3.4)	142 (4.6)	1.4

## Data Availability

This article includes all aggregated statistical results. Patient-level data used in this study are not publicly available.
